# Yan-Hou-Qing formula attenuates ammonia-induced acute pharyngitis in rats via inhibition of NF-κB and COX-2

**DOI:** 10.1186/s12906-020-03077-1

**Published:** 2020-09-14

**Authors:** Min Xu, Tian-Yong Hu, Dong-Cai Li, Li Ma, Hua Zhang, Jun-Ting Fan, Xiao-Mei Fan, Xian-Hai Zeng, Shu-Qi Qiu, Zhi-Qiang Liu, Bao-Hui Cheng

**Affiliations:** 1 Department of Otolaryngology, Longgang E.N.T hospital & Shenzhen Key Laboratory of E.N.T, Institute of E.N.T, 3004 Longgang Avenue, Shenzhen, 518172 China; 2 Zunyi Medical University, Zunyi, 563000 Guizhou China; 3 Department of Otolaryngology, The third hospital of Mianyang, Mianyang, 621000 China; 4 Department of Pharmaceutical Analysis, School of Pharmacy, Nanjing Medical University, Nanjing, 211166 China; 5 Baoan Maternal and Child Health Hospital, Jinan University, Shenzhen, 518102 China

**Keywords:** Pharyngitis, Inflammation, Yan Hou Qing, NF-κB; COX-2

## Abstract

**Background:**

Yan Hou Qing (YHQ) is a Chinese medicinal formula designed to alleviate sore throat symptoms, but underlying mechanism of YHQ treatment for pharyngitis is poorly defined up to now.

**Methods:**

In this study, the modulation of YHQ on pharyngitis is investigated in ammonia-induced acute pharyngitis rat models. After treatment with YHQ or dexamethasone respectively for five consecutive days, all rats were sacrificed for biomolecular and histopathologic study. Protein expressions of MAPKs, NF-κB, COX-2 and 5-LOX in pharyngitis tissue were evaluated by western blot analysis and the levels of TNF-α, IL-6, prostaglandin (PG) E_2_, leukotrienes (LT)-B_4_ and LT-D_4_ in pharyngeal tissue were measured via ELISA assay. Evans blue (EB) dye exudation test was performed parallelly to assess the integrity of pharyngeal tissue.

**Results:**

Compared with normal control group, EB dye exudation, and inflammatory cytokines in the model group were significantly increased, and the pharynx tissue was obviously infiltrated by inflammatory cells. YHQ treatment improved the inflammatory infiltrate in pharyngeal tissue, and reduced EB dye exudation in AP rat models. The up-regulated TNF-α and IL-6 in pharyngeal tissue of AP were significantly reduced by YHQ through inhibition of phosphorylation of p38, Erk and NF-κB. YHQ treatment also reversed the increased level of PGE_2_ through down-regulation of COX-2.

**Conclusions:**

YHQ formula attenuated the pharyngitis related symptoms via suppression of COX-2 and phosphorylation of p38, Erk and NF-κB (p65).

## Background

Pharyngitis, characterized by itchy throat and cough, is a common upper respiratory tract disease [1, 2]. It is estimated that about 600 million cases of symptomatic pharyngitis occur annually worldwide, and pharyngitis prevalence is up to 20% among children aged 0–14 years in China [3, 4]**.** It causes enormous use of health resources and the total cost of pharyngitis among children ranges from $224 to $539 million per year in the United States [5]. Pharyngitis can be caused by virus, bacteria, smoking, allergic allergen and acid reflux, and antibiotics is recommended for group A streptococcus (GAS)-caused pharyngitis therapy. Clinical management of non-infection pharyngitis remains a matter for debate because of the poor therapeutic effect of antibiotics [6]. It has been well documented that inflammatory responses play a key role in the pathogenesis of non-infection pharyngitis, and repetitive and acute inflammatory insults may render the chronic ear-nose-throat disease and nasopharyngeal mucosa susceptible to carcinogenesis [7]. So therapeutic interventions against inflammatory may enable protection and treatment non-infection pharyngitis.

Chinese medicine has a long history in treatment of non-infection pharyngitis and attracts much attention of medical worker for its satisfactory therapeutic effect [8–10]. Yan-Hou-Qing formula (YHQ) is developed for treating respiratory diseases such as laryngopharyngitis by Shenzhen Institute of Ear Nose & Throat (ENT) and Longgang ENT hospital based on clinical experiences [11, 12]. Clinical trials for YHQ have been conducted between 2009 and 2014, and YHQ is under the application for approval of China Food and Drug Administration (CFDA) to market a new drug [13, 14]. This formula is composed of 14 species of medicinal herbs (detailed information see Table 1). Our previous in vitro study revealed that YHQ possess anti-inflammatory activity in lipopolysaccharide (LPS)-stimulated murine macrophages via inhibition of nuclear factor (NF)-κB activity, and in vivo study demonstrated that YHQ attenuate allergic airway inflammation through upregulation of regulatory T cell (Treg) and suppressing Th2 responses in Ovalbumin-induced asthmatic mice [15, 16]. However, underlying mechanisms of YHQ on pharyngitis have not been clarified clearly. Ammonia-induced acute pharyngitis (AP) rat model is a non-infection pharyngitis rat model prevalently used for assessment of the therapeutic effect of complementary and integrative medicine on non-infection pharyngitis. Thus, the aim of present study was designed to determine the underlying mechanisms of YHQ on non-infection pharyngitis based on ammonia-induced AP rat models.

**Table 1:** Formula of YHQ

Formula YHQ	Amount	Formula YHQ	Amount
*L. japonica* Thunb.	125 g	*S. tonkinensis* Gagnep.	25 g
*S. ningpoensis* Hemsl.	125 g	*B. chinensis* (L.) DC.	25 g
*C. album* (Lour.) DC.	125 g	*T. sagittata* Gagnep.	25 g
*S. lychnophora* Hance	125 g	*P. alkekengi* L.	25 g
*T. chebula* Retz.	125 g	*F. cirrhosa* D. Don	25 g
*P. grandiflorum* (Jacq.) A. DC.	125 g	*C. camphora* (L.) Presl	2.5 g
*S. grosvenorii* (Swingle) C. Jeffrey ex A.M.Lu & Zhi Y.Zhang	125 g	*M. canadensis* L.	1.5 g

## Methods

### Preparation and quality control of YHQ formula

The botanical origins of these herbal medicines were authenticated by Dr. Yi-Sheng Li, Shenzhen Key Laboratory of ENT, Institute of ENT & Longgang ENT hospital, Shenzhen, China. All voucher specimens (detailed numbers mentioned in following production procedure) were deposited in the specimen room of Shenzhen Key Laboratory of ENT, Institute of ENT, Shenzhen, China.

The production procedures of YHQ formula were performed by Guangzhou Kangyuan Pharmaceutical Co., Ltd. (Guangzhou, China) according to the standard operating procedures. Briefly, *Lonicera japonica* Thunb. (No.2013110201), *Scrophularia ningpoensis* Hemsl. (No.2013110202)*, Canarium album* (Lour.) DC. (No.2013110203), *Sterculia lychnophora* Hance (No.2013110204), *Terminalia chebula* Retz. Hance (No.2013110205) and *Siraitia grosvenorii* (Swingle) C. Jeffrey ex A. M. Lu & Zhi Y. Zhang Hance (No.2013110206) were extracted with 7.5 L boiling water for 2 h for twice, and then concentrated under reduced pressure at 60 °C. The extract solution of *Platycodon grandiflorum* (Jacq.) A. DC. (No.2013110207), *Belamcanda chinensis* (L.) DC. (No.2013110208), *Sophora tonkinensis* Gagnep (No.2013110209), *Tinospora sagittata* Gagnep. (No.2013110210) and *Physalis alkekengi* L. (No.2013110211) were obtained with aqueous ethanol refluxing using 60% EtOH (1 L × 3, 1 h each time). These two extracts were combined and further spray dried with hot air at 150 °C, and the herbal extract ratio was about 23.3%. Spray dried extract mixed with powder of *F. Cirrhosa* D. Don (No.2013110212) with addition of powder sweetening agent, and was further granulated spraying with ethanol solution of *M. canadensis* L. (No.2013110213) and *C. camphora* (L.) Presl. (No.2013110214). After tablet coating procedures, the YHQ formula was finally obtained.

**Fig. 1:**
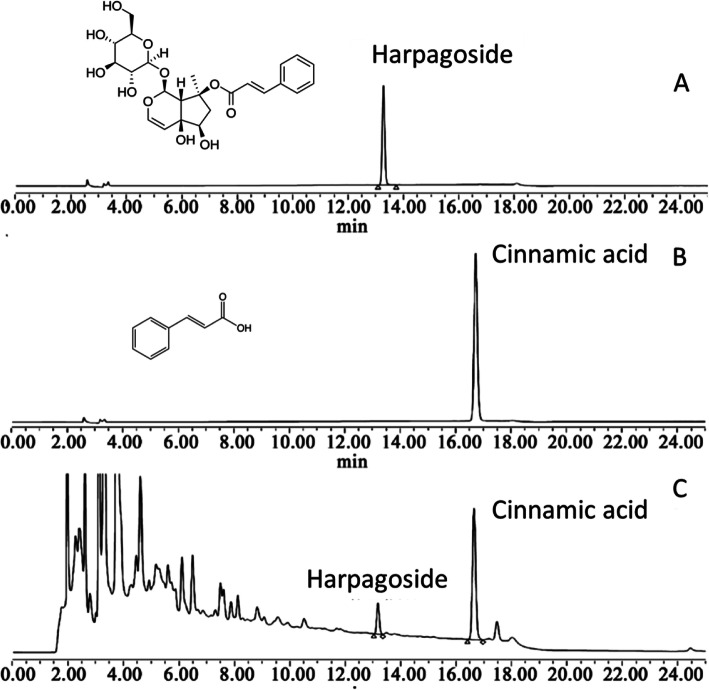
Representative HPLC-DAD chromatogram (at 279 nm) of the chemical standard of harpagoside (**a**), cinnamic acid (**b**) and YHQ formula (**c**)

Product quality was monitored by high performance liquid chromatography (HPLC). In brief, harpagoside, and cinnamic acid, as the main anti-inflammation components in *S. ningpoensis* Hemsl. was selected as the chemical markers for quality control [17–19]. Harpagoside and cinnamic acid were prepared in methanol in series of concentrations. 10 tablets of YHQ formula (batch numbers: 140401, 140,403, 140,403, 140,501, 140,502, 140,503, 140,601, 140,602, 140,603) were ground into powder, extracted with methanol and fixed to 5 ml in volumetric bottle. All the samples were analyzed using the Waters HPLC 2695 system with photodiode array detector (Waters Corporation, MA, USA) with Waters Symmetry C18 column (250 mm × 4.6 mm i.d., 5 μm). The mobile phases consisted of acetonitrile (A) and 0.3% H_3_PO_4_ (B) in a gradient elution: 20–40% A, 0–15 min; 40–20% A, 15–15.1 min; 20% A, 15.1–25 min. The flow rate was 1.0 ml/min and the column temperature was set at 30 °C. Ten μl of sample was injected and the analytes were monitored with a photodiode array detector (PAD) at the wavelength of 279 nm. The content of harpagoside and cinnamic acid in YHQ formula were 205.5 μg/g (RSD: 0.34%) and 603.6 μg/g (RSD: 0.29%), respectively (Fig. 1) [15, 16].

### Chemicals and reagents

Formamide was provided by Sigma-Aldrich (Guangzhou, Guangdong, China). Cell lysis buffer was ordered from Beyotime Biotechnology (Shanghai, China). Antibodies for β-actin, cyclooxygenase 2 (COX-2), 5-Lipoxygenase (LOX), Erk, Phospho-Erk, JNK, Phospho-JNK, p38, Phospho-p38, IKKβ, Phospho-IKKβ, IκBα, Phospho-IκBα (Ser32/36), NF-κB p65, and phospho-NF-κB p65 were purchased from Cell Signaling Technology, Inc. (Danvers, MA, USA). Relative peroxidase-conjugated secondary antibody, Bicinchoninic acid (BCA) assay, Evans blue (EB) dye and Pierce™ ECL Western Blotting Substrate were purchased from Thermo Scientific (Waltham, MA, USA). ELISA for IL-6, TNF-α, PGE_2_, LT-B_4_ and LT-D_4_ were purchased from Cusabio (Wuhan, Hubei, China).

### Experimental animals

Female SD rats (180–220 g) from Guangdong experimental animal center were used for the study. The animals were maintained at 25 °C in a clean environment under 12: 12 h light–dark cycle. All rats had free access to food and distilled water. All the experimental protocols were approved by Institutional Animal Care and Use Committee (IACUC) at Shenzhen Institute of ENT (SZENT-2018-012).

### Induction of pharyngitis and animal treatment

**Fig. 2:**
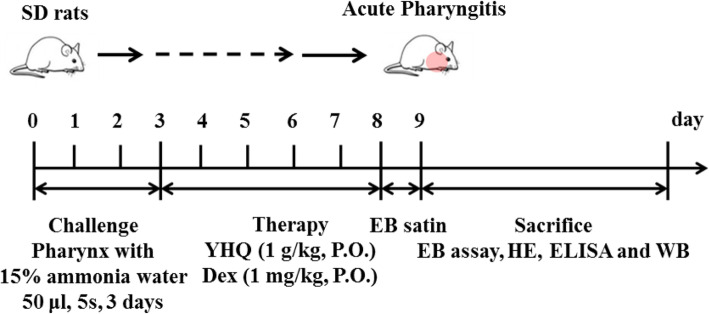
Schematic diagram of acute pharyngitis induction and YHQ treatment

Acute pharyngitis (AP) model was established by spraying 15% ammonia water on the pharynx of rats from day 1 to day 3 (spray 3 thrushes every time, cause the pharynx mucosa to be hyperemia swollen, and form the acute inflammation) and normal control group (NC) was sprayed with same volume of saline. The ammonia induced pharyngitis rat models were randomly divided into AP model group (12 rats), YHQ treatment group (12 rats) and dexamethasone treatment group (Dex) (12 rats) on the 4th day. The dosage of YHQ used in ammonia-induced AP treatment is transformed from the clinical used dosage of YHQ (Based on phase I-II clinical trials study, and there is no toxicity observed when rats were oral administrated with YHQ up to dose of 8 g/kg) according to the “Guidance for Industry Estimating the Maximum Safe Starting Dose in Initial Clinical Trials for Therapeutics in Adult Healthy Volunteers” published by U.S. Food and Drug Administration (https://www.fda.gov/media/72309/download). As a steroidal anti-inflammatory drug, long-time oral use of dexamethasone reported with a variety of side effects, so on the premise of ensuring the effective dose of dexamethasone, the dexamethasone dosage should be used as low as possible in ammonia-induced AP. For the treatment group, YHQ formula and dexamethasone were oral administrated 1 g/kg and 1 mg/kg once a day for 5 days, respectively. NC group and AP group were orally given with same volume of saline (Fig. 2).

### Behavioral study and pharyngeal tissue pathological evaluation

According to behavioral and physical marking criteria (Table 2), scores for diet, activities, mouth scratch, mouth hair, cough and saliva secretion variations for ammonia induced AP rat models after YHQ and dexamethasone treatment were assessed. Scores for mucosal hyperplasia, cell infiltration, vascular dilation & bleeding and gland hypertrophy changes were also determined based on pathological marking criteria (Table 3) for all rat groups.

**Table 2:** Behavioral and physical score criteria for ammonia induced pharyngitis rat models

Symptom rating	Diet reduction	Activities reduction	Mouth scratch	Mouth hair loss	Cough	Saliva secretion	Pharynx swelling
0	No	No	Normal	No	No	No	Normal
1	Mild	Mild	Few	Few	Mild	little	Mild
2	Obvious	Obvious	Many	Many	Severe	Mass	Severe

**Table 3:** Pathological score criteria for ammonia induced pharyngitis rat models

Score criteria	Mucosal hyperplasia	Cell infiltration	Vascular dilation & Bleeding	Gland hypertrophy
0	No	No	No	No
1	Mild	Mild	Mild	Mild
2	Moderate	Moderate	Moderate	Moderate
3	Severe	Severe	Severe	Severe

### Evaluation of pharyngeal mucus integrity via Evans blue dye

Parallel to these experiments another set of experiments were run without administration of EB dye. On 8th day after administration of last dose of assigned treatments, EB dye (30 mg/kg, i.v.) was administered to all rats in NP, AP, YHQ and Dex group via tail intravenous injection. One hour later, all rats were sacrificed by exsanguination and the head portion was perfused with heparinized saline to expel the intravascular EB dye. After the removal of pharynx tissue, pharynx tissue was cut into pieces, extracted with formamide at 55 °C for 24 h and further determined at 620 nm using SpectraMax Paradigm Multi-Mode Microplate Reader (Molecular Devices, CA, USA).

### Determination of inflammatory and pro-inflammatory cytokines in pharynx

As the typical pro-inflammation cytokines (TNF-α and IL-6) and inflammation mediators (PGE_2_, LT-B_4_ and LT-D_4_) indicating degree of inflammation in ammonia-induced acute pharyngitis rat model, these pro-inflammation cytokines and inflammation mediators in pharyngitis tissue were detected [20]. After homogenization and centrifugation, concentrations of TNF-α, IL-6, PGE_2_, LT-B_4_ and LT-D_4_ in supernatant were measured by quantitative sandwich enzyme-linked immunoassay (ELISA) kits (CUSABIO, Wuhan, Hubei, China). All ELISA experiments were strictly performed according to the manufacture’s instruction. Absorbance was measured at 450 nm using SpectraMax Paradigm Multi-Mode Microplate Reader (Molecular Devices, CA, USA).

### Western blot analysis of inflammation-related enzymes

As the key proteins involved in triggering inflammation, the protein expressions of arachidonate 5-lipoxygenase (5-LOX), cyclooxygenase 2 (COX-2), Erk, phospho-Erk, JNK, phospho-JNK, p38, phospho-p38, IKKβ, phospho-IKKβ, IκBα, phospho-IκBα, NF-κB p65, phospho-NF-κB p65 were probed via western blot [21]. To perform protein extraction, pharyngeal tissues were homogenized and extracted with cell lysis buffer with protease and phosphatase inhibitor cocktail. The protein concentrations were determined by BCA assay. Protein samples were then separated by SDS-PAGE containing 8% of SDS, and electroblotted onto PVDF membranes (GE Healthcare, USA). The membranes were blocked with 5% BSA solution (in Tris-buffered saline (150 mM NaCl, 20 mM Tris–HCl, pH 7.6) with 0.1% Tween 20) for 1 h at room temperature. The membranes were then incubated with primary antibodies against 5-LOX, COX-2, Erk, phospho-Erk, JNK, phospho-JNK, p38, phospho-p38, IκB kinase (IKK) β, phospho-IKKβ, IκBα, phospho-IκBα, NF-κB p65, phospho-NF-κB p65 and β-actin at 4 °C overnight. After washing with Tris buffered saline, peroxidase-conjugated secondary antibodies were added to the membranes and incubated at room temperature for 1 h. The expressions of proteins were detected by ECL reagent, followed by exposure of the membranes to ChemiDoc™ MP Imaging System (Bio-Rad, Hercules, California, USA). The intensity of protein bands was measured by densitometry and quantified using the Image J Software.

### HE and immunofluorescence staining

Pharynx tissue was infused by 4% paraformaldehyde fixing solution and the specimens were dehydrated and embedded in paraffin. For histological examination, 4 μm sections of embedded tissues were cut using a Leica RM2235 rotary microtome (Wetzlar, Germany), placed on glass slides, deparaffinized, and stained sequentially with hematoxylin and eosin (HE). Images of lung tissue sections stained with HE were acquired with a Nikon Eclipse Ci-L/S microscope (Nikon, Tokyo, Japan) equipped with a digital imaging system (Nikon, Tokyo, Japan).

### Immunofluorescence and confocal microscopy

For immunofluorescence staining, pharynx tissue slides were permeabilized with PBS containing 0.3% Triton X-100 and 10% BSA. Sections of tissues were incubated with NF-κB p65 primary antibodies overnight at 4 °C and then incubated with FITC labeled secondary antibodies in the dark for 1 h at 25 °C. Cell nuclei were stained using DAPI for 10 min, and fluorescent images were captured with LEICA TCS SP5 II confocal microscope (Leica Microsystems Inc. Buffalo Grove, IL, USA).

### Statistical analysis

Behavioral and physical scores and pathological scores were exhibited as median with interquartile rage, and other results were expressed as mean ± SEM. Each experiment was repeated thrice in triplicate. Multiple group comparisons were performed using one-way ANOVA followed by Tukey test using GraphPad Prism 5.01 (La Jolla, CA, U.S.A.). The differences were considered as statistically significant when *p* < 0.05.

## Results

### Pathological improvement of YHQ on pharynx in AP rat models

**Fig. 3:**
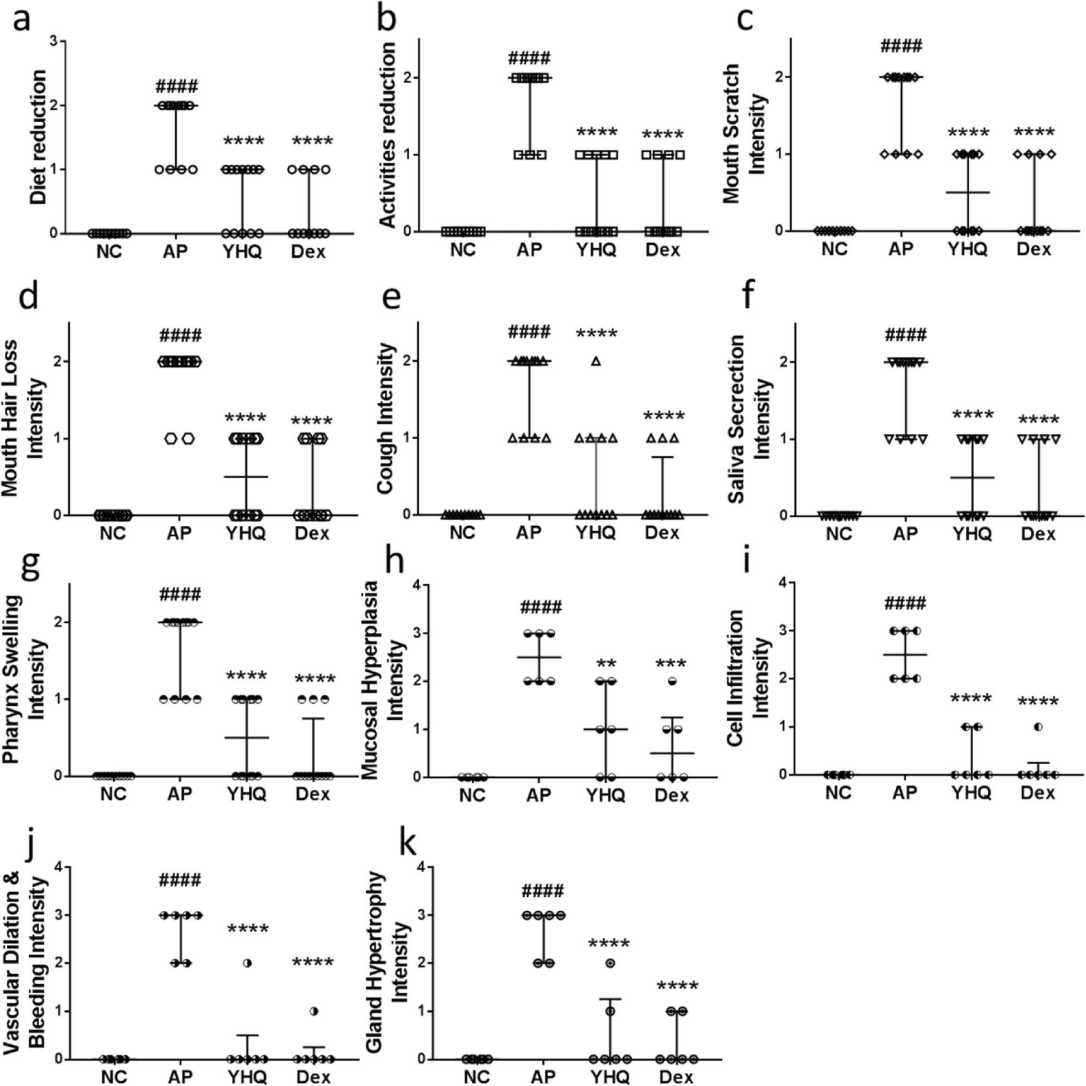
Modulation of YHQ on behaviors (**a**-**f**) and pharynx pathology (**e**-**k**) in ammonia-induced acute pharyngitis. Data are shown as the median with interquartile rage. Statistical analysis were conducted via one-way ANOVA followed by a Tukey’s Multiple Comparison test (^####^*p* < 0.0001 vs. NC, ** *p* < 0.01, *** *p* < 0.001, **** *p* < 0.0001 vs. AP)

After modeling, the frequencies of mouth scratch and cough increased, and food intake behaviors decreased (details see Fig. 3). Pharynx showed redness and swelling and accumulated mucous secretions, and superficial ulcer was formed. Some of the AP rat models lost their mouth hair gradually. The normal control group had normal autonomic activity, and there was no ulceration in pharyngeal mucosa.

According to the pharyngitis pathological score criteria, the rat pharynx tissues in each group were assessed (Tables 2 and 3). As shown in Fig. 3, the pathologies of the pharyngeal mucosa and tissue were changed significantly (*p* < 0.0001) in the model group. In model group, the mucous membranes of the pharyngeal tissue of rat form ulcers, and hyperemia and infiltration of inflammatory cells can be seen under the mucous membranes of ulcers. Pharyngeal inflammatory reaction of rats in the NC group show no the appearance of inflammation, and mucosal and submucosal mucosal glands of the pharyngeal tissue of rats were normal. The inflammatory symptoms of pharyngeal tissue in each YHQ group and Dex group improved in different degrees.

### YHQ reduces EB dye lesion in pharyngitis tissue via maintaining mucosal integrity

**Fig. 4:**
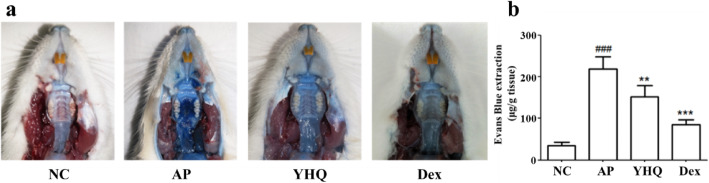
Effect of YHQ on ammonia-induced morphological damage of rat pharynx. **a** Evans Blue (EB) dye lesion due to morphological damage of rat pharynx; **b** Variations of EB dye in pharyngeal tissue. Data are shown as the mean ± SEM. Statistical analysis were conducted via one-way ANOVA followed by a Tukey’s Multiple Comparison test (^####^*p* < 0.0001 vs. NC; ** *p* < 0.01, *** *p* < 0.001 vs. AP)

On 8th day, 1 h after administration of EB dye (30 mg/kg, i.v.) the pharyngeal tissue was separated and the quantity of EB dye present in the pharyngeal tissue was quantified by using standard curve for EB dye (Fig. 4). Dexamethasone and YHQ treated animals showed macroscopic reduced blue ting, as an indication of their protective effect against ammonia-induced damage (Fig. 4a). AP group showed severe extravasation of EB dye due to inflammation of pharynx, however NC animals applied with saline showed almost no extravasation of EB dye (Fig. 4b). The severe extravasation EB dye levels of pharyngeal tissue were attenuated significantly after 5 days’ treatment with YHQ and dexamethasone (*p* < 0.01 and *p* < 0.001, respectively).

### YHQ attenuates the cell infiltration in ammonia induced pharyngitis rat model

**Fig. 5:**
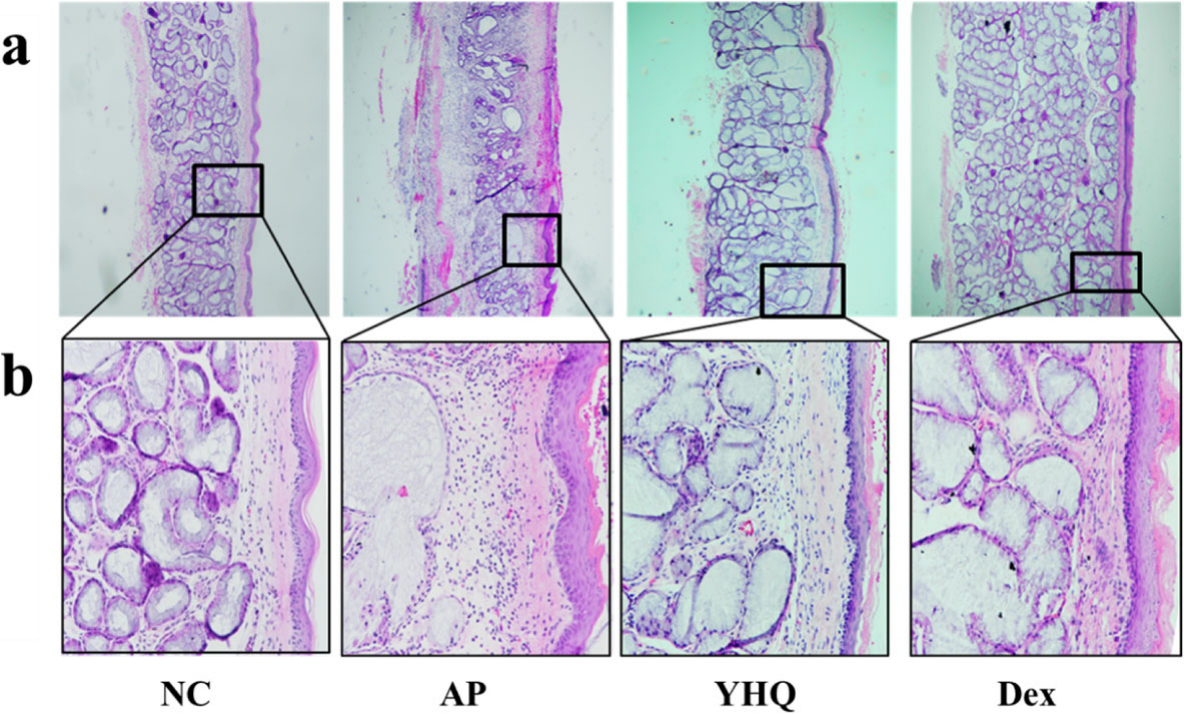
The Effect of YHQ on ammonia-induced pharyngitis in rats. Pharynx tissue slices were fixed, embedded, and stained with HE. Microscope images were displayed at a magnification of 40 × (**a**) and 400 × (**b**)

Histopathological changes on pharynx tissue were assessed by HE staining. H&E results showed of submucosal gland hypertrophy and thickened mucosa in pharynx of AP rats (Fig. 5), and NC rats showed normal cytoarchitecture of the pharynx. Dexamethasone (1 mg/kg, p.o.) treated animals showed mild hypertrophy of mucus glands and thin mucosa, especially in improving hypertrophy of mucus gland. Compared to Dex group, YHQ (1 g/kg, p.o.) treated animals showed milder hypertrophy of mucus glands and thinner mucosa which was similar to the NC group (Fig. 5). Noteworthy, YHQ (1 g/kg, p.o.) were found to be more potent than reference standard dexamethasone (1 mg/kg, p.o.) in reducing submucosal gland hypertrophy and mucosa thickness in AP rats.

### Down-regulation of YHQ on proinflammatory cytokines in pharyngitis rat models

**Fig. 6:**
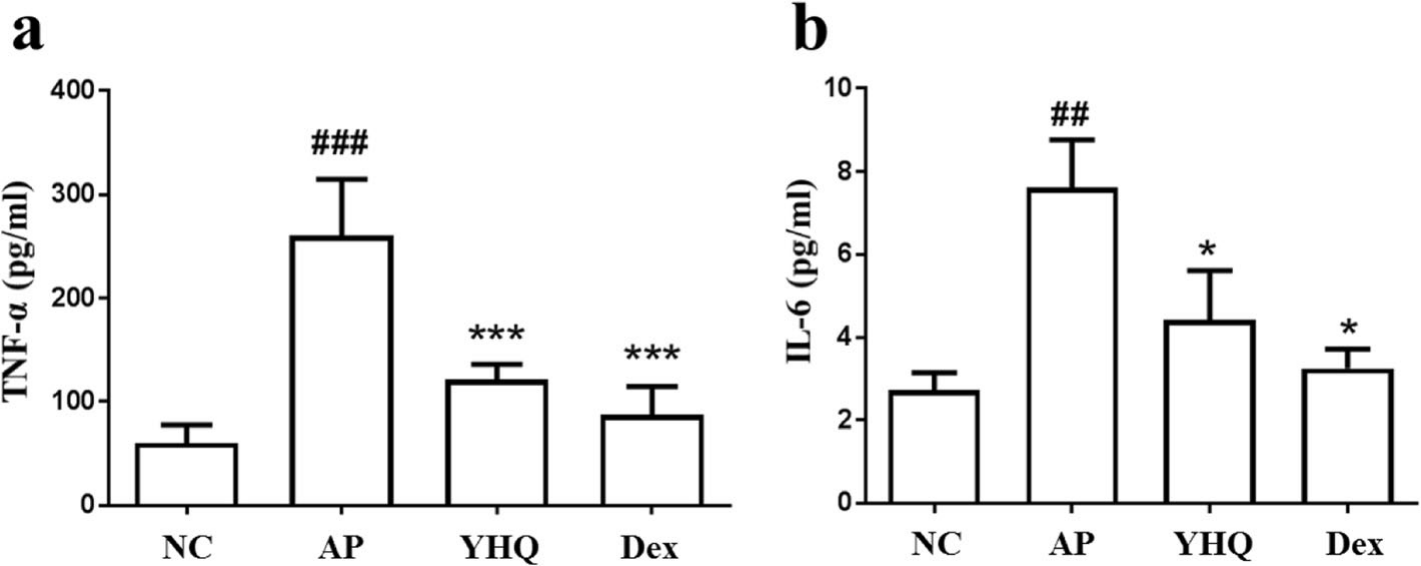
The effects of YHQ on TNF-α and IL-6 levels in pharynx tissue of rats. (**a**) Levels of TNF-α; (**b**) Levels of IL-6. Data are shown as the mean ± SEM. Statistical analysis were conducted via one-way ANOVA followed by a Tukey’s Multiple Comparison test (^##^*p* < 0.01, ^###^*p* < 0.001 vs. NC; *p < 0.05, ***p < 0.001 vs. AP)

As pro-inflammatory cytokines, interleukin (IL)-6 and tumor necrosis factor (TNF)-α are involved in acute inflammation and make up the acute phase reaction [22]. Compared with the normal control group, the levels of TNF-α (*p* < 0.001 and *p* < 0.05, respectively, Fig. 6) and IL-6 (*p* < 0.001 and *p* < 0.05, respectively, Fig. 6) in model group increased significantly, and this up-regulations of TNF-α (*p* < 0.001, *p* < 0.01, Fig. 6) and IL-6 (*p* < 0.001, *p* < 0.01, Fig. 6) were alleviated after treatment with YHQ or dexamethasone. The inhibition of YHQ on TNF-α and IL-6 implied that alleviation of YHQ on pharyngitis may depend on down-regulation of inflammatory mediators.

### YHQ treatment reduces LT-B_4_ and LT-D_4_ in pharyngitis rat model

**Fig. 7:**
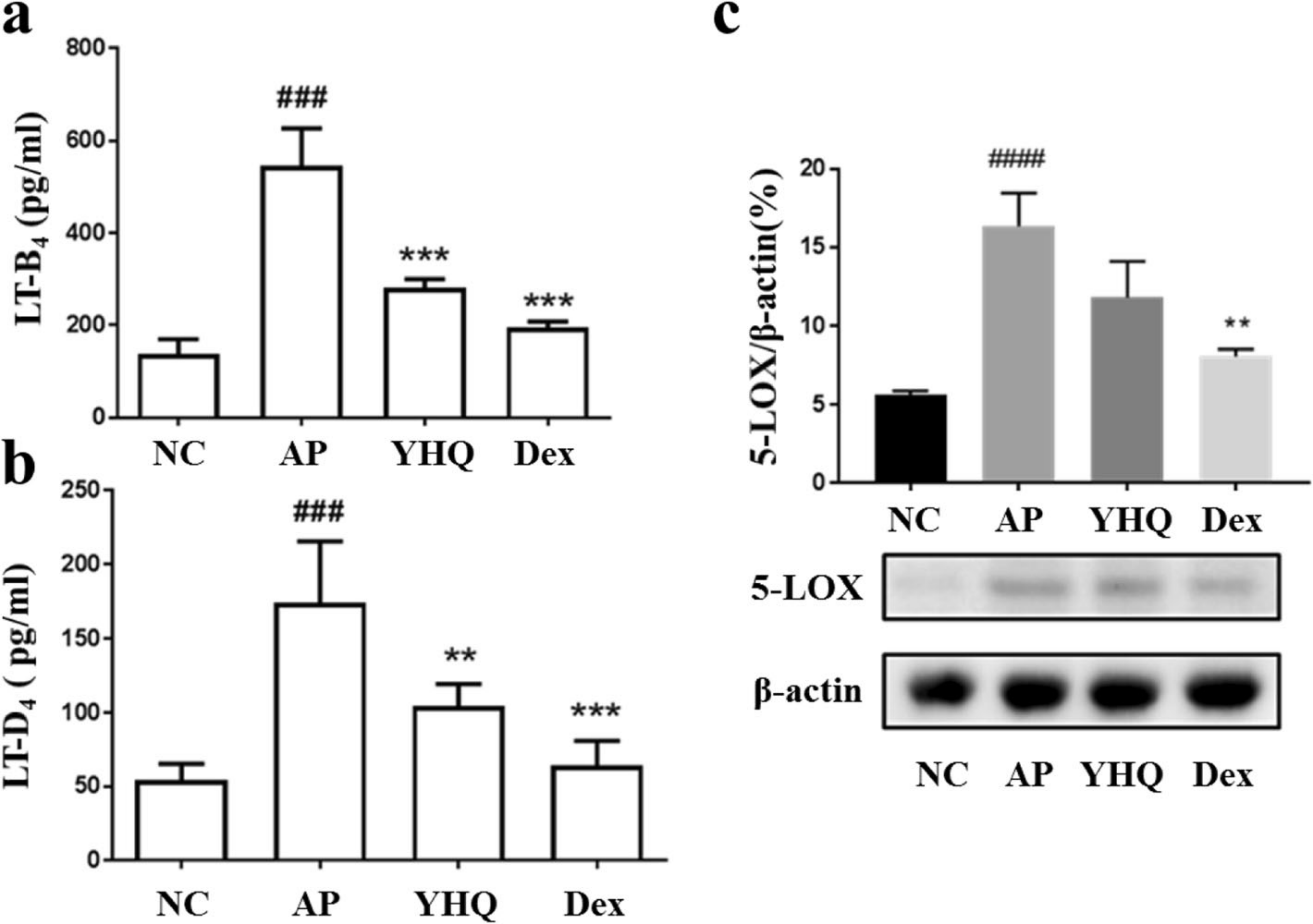
The effects of YHQ on LT-B_4_ and LT-D_4_ levels in pharynx tissue of rats. **a** Levels of LT-B4; **b** Levels of LT-D4. Data are shown as the mean ± SEM. Statistical analysis were conducted via one-way ANOVA followed by a Tukey’s Multiple Comparison test (^###^*p* < 0.001 vs. NC; **p < 0.01, ***p < 0.001 vs. AP)

For the down-regulation of YHQ on pro-inflammatory cytokines, the inhibitory effects of YHQ were further evaluated on inflammatory mediators (LT-B_4_ and LT-D_4_) and related synthesized protein 5-LOX. As a fundamental regulator in inflammation process, aiming at inhibition of 5-LOX has been deliberated as an effective approach to prevent inflammatory disorders. Effect of YHQ was evaluated on 5-LOX mediated production of LT-B_4_ and LT-D_4_ in acute pharyngitis rat model. ELISA analysis (Fig. 7a and b) showed that ammonia sprays up-regulated the release of LT-B_4_ and LT-D_4_ in pharynx which was significantly attenuated after treatment with YHQ at a dose of 1 g/kg (*p* < 0.001 and *p* < 0.01, respectively). As the synthesis protein for LT-B_4_ and LT-D_4_, the expressions of 5-LOX in pharynx tissue were investigated by western blot, and 5-LOX protein was considerably upregulated in the ammonia induced pharyngitis compared to normal control group. As illustrated in Fig. 6c, the expression of 5-LOX stimulated by ammonia spray, were significantly suppressed after treatment with dexamethasone (*p* < 0.01, Fig. 7c). Compared to AP group, after the treatment with YHQ at 1 g/ml, and the 5-LOX protein expressions were decreased by 4.5%, and YHQ show no significant inhibition on 5-LOX (*p* > 0.05, Fig. 7c).

### Suppression of YHQ on PGE_2_ via reduction of COX-2 expression

**Fig. 8:**
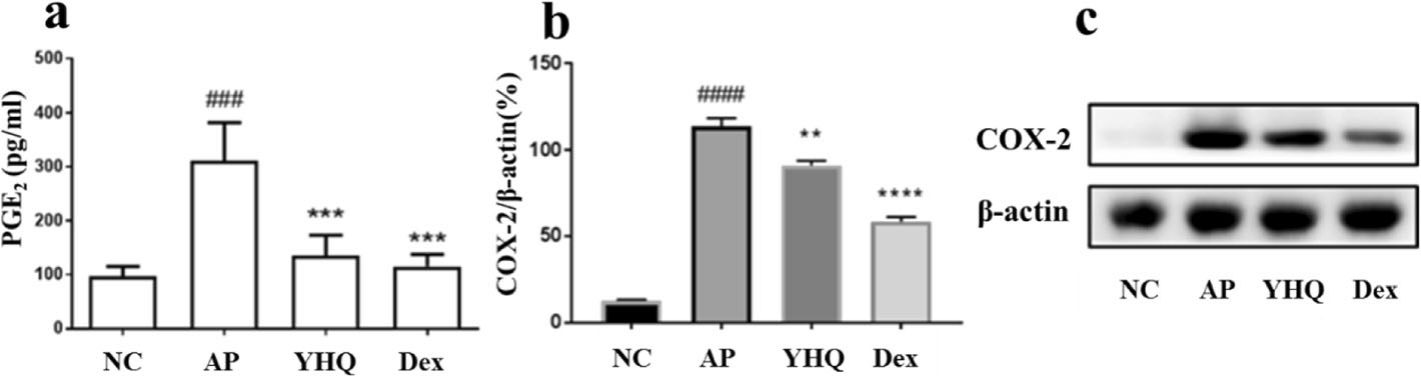
The effects of YHQ on PGE2 levels and COX-2 expression in pharynx tissue of rats. Data are shown as the mean ± SEM. Statistical analysis were conducted via one-way ANOVA followed by a Tukey’s Multiple Comparison test (^###^*p* < 0.001 vs NC; ***p < 0.001 vs. AP)

PGE_2_, an arachidonic acid derivative generated by cyclo-oxgenase-2 (COX-2), is another principal mediator of acute inflammation. ELISA was performed to investigate whether YHQ suppress PGE_2_ production in ammonia-induced pharyngitis. YHQ was found to decrease the level of PGE_2_ significantly at dose of 1 g/kg (Fig. 8a). As shown in Fig. 8b and c, YHQ significantly reduced COX-2 expression (*p* < 0.01) at dose of 1 g/kg. Compared to pharyngitis group, after the treatment with YHQ at 1 g/kg, and the COX-2 protein expression was decreased by 22.7%. This result suggests that suppression of YHQ on PGE_2_ production is related to the regulation of the expression of its synthesis enzyme COX-2 in ammonia-induced pharyngitis.

Nevertheless, these outcomes recommend that the inhibitory effects of YHQ on 5-LOX/LT-B_4_/LT-D_4_ and COX-2/PGE_2_ are consistent with its inhibitory effects on pro-inflammatory cytokines (IL-6 and TNF-α) in ammonia-induced pharyngitis.

### Impact of YHQ on MAPKs and NF-κB signaling pathway mediated inflammation in ammonia-induced pharyngitis

**Fig. 9:**
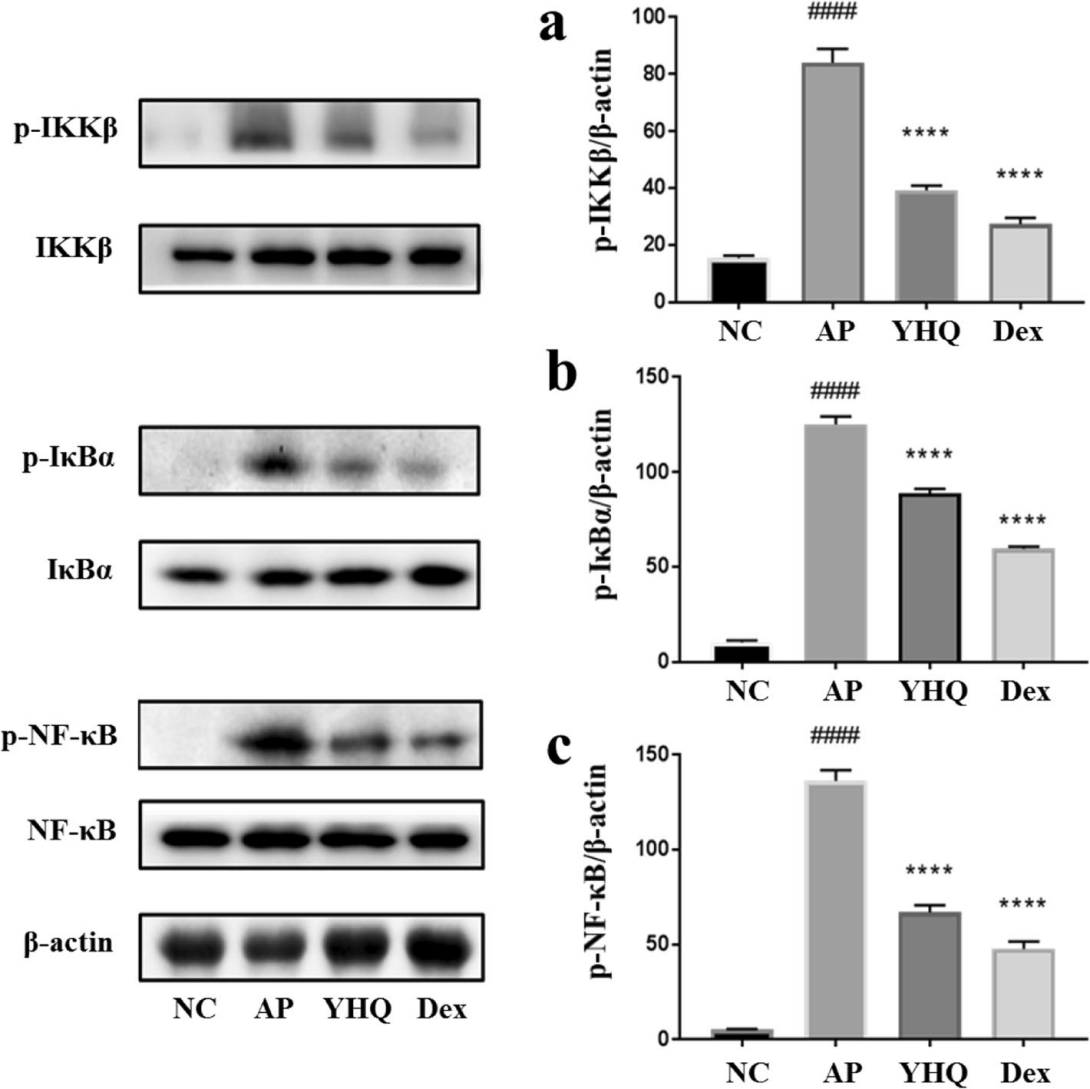
Effects of YHQ on phosphorylated IKKβ (p- IKKβ), p-IκBα and p-NF-κB expression in pharynx tissue of rats. Data are shown as the mean ± SEM. Statistical analysis were conducted via one-way ANOVA followed by a Tukey’s Multiple Comparison test (^####^*p* < 0.0001 vs. NC; ****p < 0.0001 vs. AP)

**Fig. 10:**
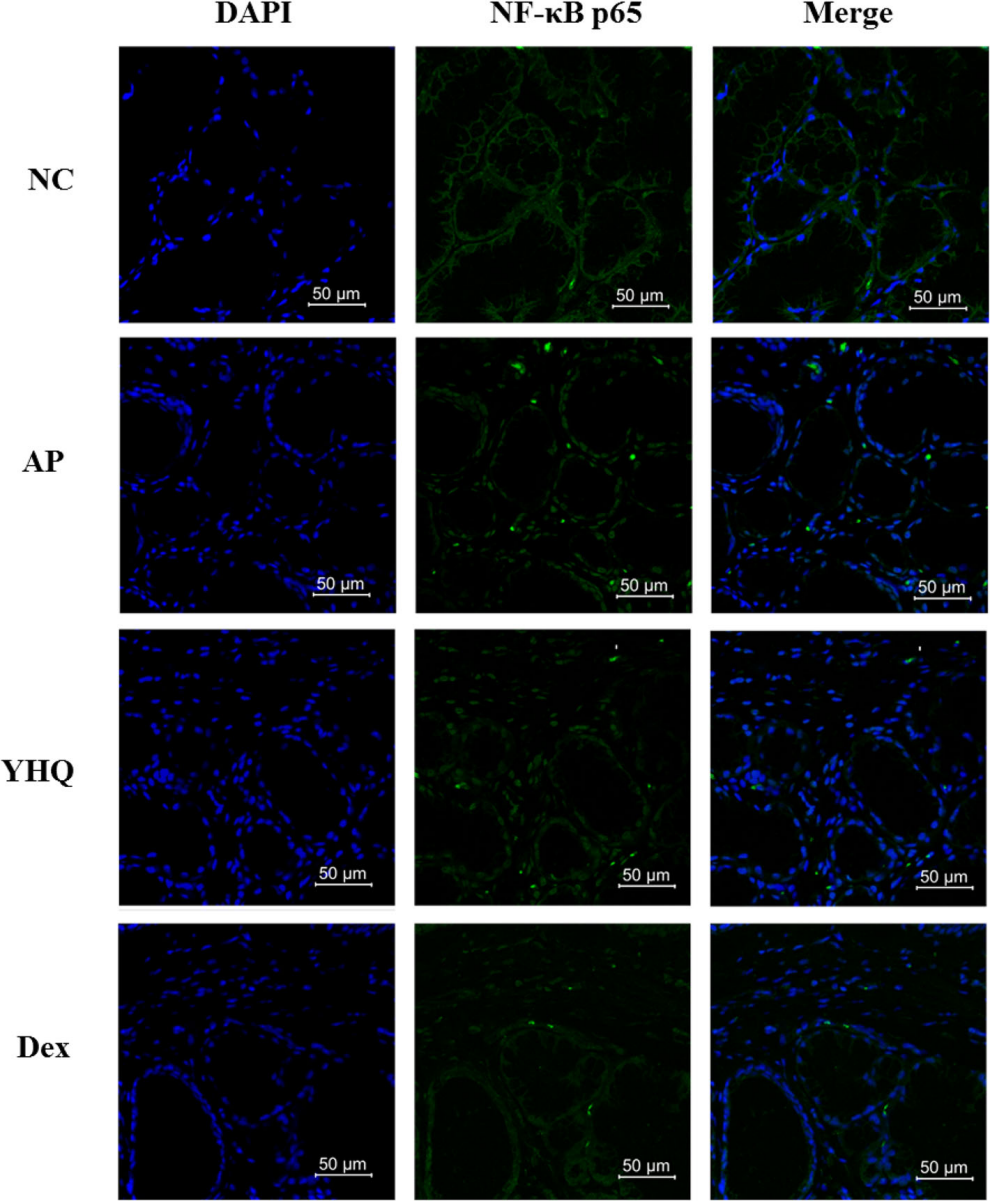
The translocation of p65 to the nucleus was analyzed by confocal microscopy. Cells were immunostained using FITC for NF-κB p65 and DAPI for nucleus

Tissue damages through the intracellular signaling cascade activates a trimeric IKK complex and then phosphorylates IκBα resulting in nuclear translocation of the phosphor-NF-κB for further the transcription of the genes encoding pro-inflammatory cytokines and chemokines [23, 24]. The IKK enzyme complex is a part of the upstream NF-κB signal transduction cascade, so YHQ was evaluated for its effect on pIKKβ/NF-κB signaling molecules including NF-κB (p65), p-NF-κB (p65), IκBα, p-IκBα, IKKβ and p-IKKβ in pharyngeal mucous tissue of ammonia induced pharyngitis. As compared to the NC group, stimulation with ammonia enhanced the phosphorylation of p65, p-IκBα and IKKβ (Fig. 9). Treatment with YHQ showed significant inhibition on p-NF-κB (p65) in respect to ammonia-induced pharyngitis (*p* < 0.0001 for 1 g/kg, Fig. 9c). The modulation of YHQ on NF-κB signal transduction in ammonia-induced pharyngitis rat model depended on prevention of YHQ on p-IκBα and p-IKKβ. Suppression of YHQ on p-NF-κB (p65) was via inhibiting p-IKKβ and p-IκBα upregulated by ammonia stimulation (*p* < 0.001 and *p* < 0.001, respectively) without interfering the level of IκBα, IKKβ and NF-κB p65 in rat models (Fig. 9a and b). We also confirmed this result via confocal microscope and immunofluorescence results indicated that fluorescence intensity in cell nuclei of pharynx induced by ammonia is apparently suppressed by YHQ suggesting that YHQ down-regulated the expression of NF-κB-regulated genes at the transcriptional level (Fig. 10).

**Fig. 11:**
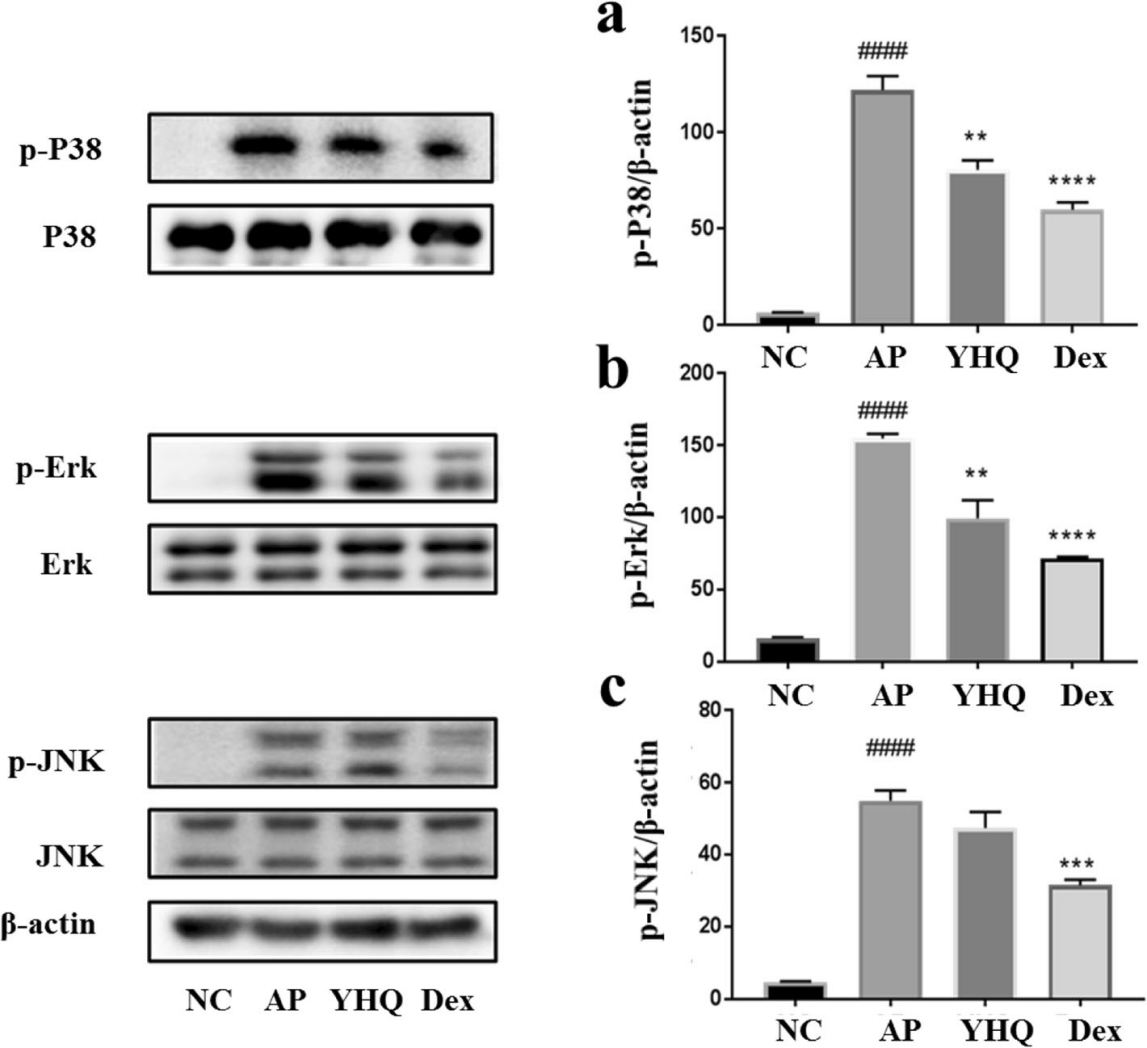
Effects of YHQ on phosphorylated p38 (p-p38), p-Erk and p-JNK expression in pharynx tissue of rats. Data are shown as the mean ± SEM. Statistical analysis were conducted via one-way ANOVA followed by a Tukey’s Multiple Comparison test (^####^*p* < 0.0001 vs. NC; **p < 0.01, ***p < 0.001, ****p < 0.0001 vs. AP)

The MAPK signaling cascades are well-thought-out to be involved in this molecular mechanism [25, 26]. During inflammatory processes, phosphorylation of MAPKs (JNK, ERK, and p38) implement critical role by triggering the regulation of NF-κB signaling pathway. As shown in Fig. 11, ammonia stimulation enhanced phosphorylation of Erk, JNK and p38 protein kinases, and YHQ treatment at dose of 1 g/kg inhibits the ammonia induced phosphorylation levels of p38 and Erk in acute pharyngitis rat models, while there is no significant change in protein expression of JNK and p-JNK (Fig. 11c).

## Discussion

This study assessed the effects YHQ formula on pharyngitis via ammonia induced AP rat models, and our data showed that YHQ relieve pharyngitis related symptoms, such diet reduction, activities reduction, mouth scratch, mouth hair loss, cough, saliva secretion, pharynx swelling (Fig. 3a - f), and YHQ also attenuate mucosal hyperplasia, cell infiltration, vascular dilation and gland hypertrophy (Fig. 3g - k). These results indicated that YHQ could effectively improve ammonia induced acute pharyngitis in rat model. YHQ lowered histopathology scores in pharynx, showing that YHQ could reduce the occurrence of hypertrophy gland and mucosa thickness, and relieve ammonia induced pharynx related symptoms.

Our data show that reverse the up-regulated proinflammatory cytokines TNF-α, IL-6 induced by tissue injury due to ammonia sprays. Treatment with YHQ could decrease the expressions of COX-2 and 5-LOX to further reduce the production of inflammatory cytokines PGE_2_, LTB_4_ and LTD_4_. As the main regulator of proinflammation and inflammation cytokines production (e.g. TNF-α, IL-6, PGE_2_, LTB_4_ and LTD_4_), the activation of NF-κB is a hallmark of host cells responding to ammonia induced tissue injury. Current study demonstrated that YHQ could attenuate inflammation caused by tissue injury in pharyngitis rat model via inhibiting p38/Erk/NF-κB/COX-2 signaling pathway.

The main mechanisms for current available single compound, herbal extracts and herbal formula for AP treatment are mainly involved in the anti-inflammation [27–29] . YHQ formula was designed by otorhinolaryngology physicians based on traditional Chinese medicine theory and clinic experiences, and YHQ was used as hospital preparation in form of buccal tablet at clinics. It can not only play a direct anti-inflammatory effects, but also benefit the damaged pharyngeal tissue repair. Based on phase I-II clinical trials study, and there is no toxicity observed when rats were oral administrated with YHQ up to dose of 8 g/kg. It contains 14 kinds of herbal medicines, and individual herbs involved in YHQ have been documented to have various biological effects [15]. *Lonicera japonica* Thunb. (Jin-Yin-Hua) and *Scrophularia ningpoensis* Hemsl. (Xuan-Shen) are major components in this formula possessing anti-inflammation [30–32]. *Sterculia lychnophora* Hance (Pang-Da-Hai), *Terminalia chebula* Retz. (He-Zi), *Canarium album* (Lour.) DC. (Qing-Guo), *and Belamcanda chinensis* (L.) DC. (She-Gan) can help the above major components in treatment of pharyngitis via wound healing, antibacteria, and antioxidation to improve pharyngitis symptoms [29, 33, 34]. *Siraitia grosvenorii* (Swingle) C. Jeffrey ex A.M.Lu & Zhi Y. Zhang (Luo-Han-Guo), *Physalis alkekengi* L*.* (Jin-Deng-Long), *Tinospora sagittata* Gagnep. (Jin-Guo-Gan), *Fritillaria cirrhosa* D. Don (Chuan-Bei-Mu), *Sophora tonkinensis* Gagnep. (Shan-Dou-Gen) and *Platycodon grandiflorum* (Jacq.) A. DC. (Jie-Geng) function as assistant roles (anti-tussive, anti-inflammation, antimicrobial, analgesic and tracheobronchial relaxant) in tissue injury recover during the treatment of pharyngitis [35–40]. *Mentha canadensis* L. (Bo-He) acts as penetration enhancer promote other herbal medicine absorption and also improve the throat discomfort during pharyngitis [41]. Although YHQ showed no such strong anti-inflammatory effect as reference drug dexamethasone, combination of these 14 herbal medicines exhibited better reduced gland hypertrophy and thinner mucosa in HE staining than dexamethasone used alone and showed similar improved pharyngitis symptoms as dexamethasone.

## Conclusions

This study demonstrated that YHQ possess the remarkable anti-inflammatory effects, protect the mucosal tissue integrity, and improve the pharyngitis symptoms. The therapeutic effects of YHQ on AP are associated with the reduction of pro-inflammatory cytokines (TNF-α and IL-6) and inflammatory mediators (PGE_2_, LTB_4_ and LTD_4_) caused by injuries induced by ammonia in rat model via inhibiting phosphorylation of p38, Erk, and NF-κB (p65) and lowering the protein expressions of COX-2 and 5-LOX. Our findings support the further evaluation of YHQ as a potential drug for the treatment of non-infection AP. YHQ may have significant clinical potential as a therapeutic agent for the treatment of upper airway inflammatory conditions such as pharyngitis.

## Supplementary information

**Additional file 1.**

## Data Availability

The data used or analyzed during the current study are available from the corresponding author on reasonable request.

## Abbreviations

PGE_2_: prostaglandin (PG) E_2_
LT: leukotrienes
EB: Evans blue
YHQ: Yan-Hou-Qing formula
CFDA: China Food and Drug Administration
LPS: lipopolysaccharide
Treg: Regulatory T cell
NF-κB: Nuclear factor-κB
HPLC: high performance liquid chromatography
COX-2: cyclooxygenase 2
5-LOX: 5-Lipoxygenase
AP: Acute pharyngitis
NC: control group
Dex: dexamethasone treatment group
i.v.: intravenous
ELISA: enzyme-linked immunoassay
H&E: Hematoxylin and eosin stain
IL: interleukin
IKK: IκB kinase
