# Supplementary figures and images for: Yan-Hou-Qing formula attenuates ammonia-induced acute pharyngitis in rats via inhibition of NF-κB and COX-2
Source: BMC Complement Med Ther. 2020 Sep 14;20:280. doi: 10.1186/s12906-020-03077-1 (PMC7491126; doi:10.1186/s12906-020-03077-1)

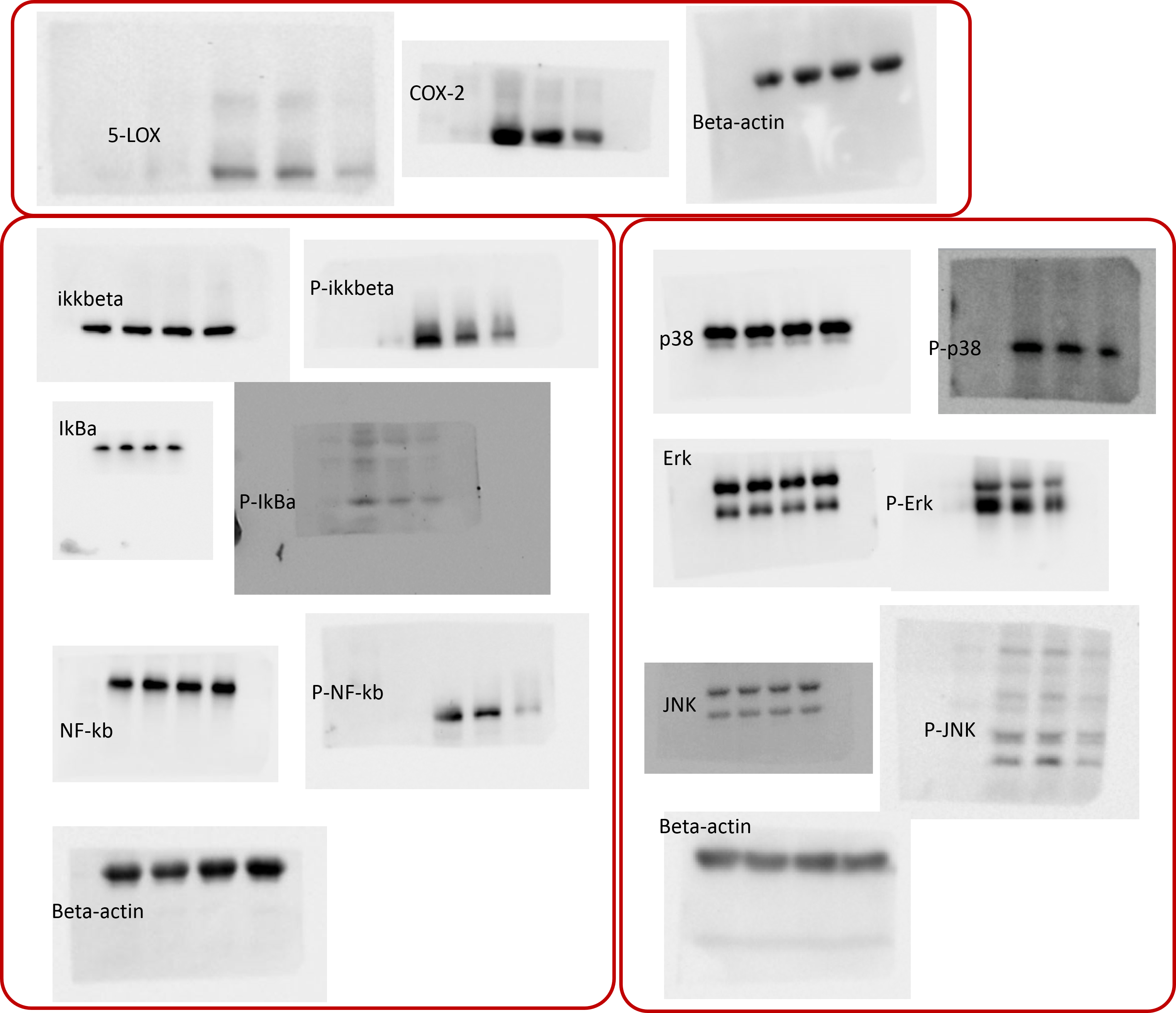

Supplement: Supplementary file 1 — Additional file 1. [file 12906_2020_3077_MOESM1_ESM.tif]
